# *De novo *deletion in *MECP2 *in a monozygotic twin pair: a case report

**DOI:** 10.1186/1471-2350-12-113

**Published:** 2011-08-27

**Authors:** Kirti Mittal, Madhulika Kabra, Ramesh Juyal, Thelma BK

**Affiliations:** 1 Genetics, University of Delhi South Campus, Benito Juarez Road, New Delhi 110021, India; 2 Pediatrics, All India Institute of Medical Sciences, New Delhi 110608, India; 3 National Institute of Immunology, Aruna Asaf Ali Marg, New Delhi, 110067, India

## Abstract

**Background:**

Rett syndrome (RTT) is a severe, progressive, neurodevelopmental disorder predominantly observed in females that leads to intellectual disability. Mutations and gross rearrangements in *MECP2 *account for a large proportion of cases with RTT. A limited number of twin pairs with RTT have also been reported in literature.

**Case Presentation:**

We investigated 13 year old, monozygotic twin females with RTT and some noticeable differences in development using a combinatorial approach of sequencing and Taqman assay. Monozygosity status of the twins was confirmed by informative microsatellite markers.

**Conclusions:**

The twins shared a *de novo *deletion in exon 3 in the MBD domain of *MECP2*. To the best of our knowledge, this is only the second report of genetic analysis of a monozygotic twin pair.

## Background

Rett Syndrome (RTT; MIM # 312750) is a severe progressive neurodevelopmental disorder that predominantly affects females. It has an estimated global prevalence of approximately one in 10,000-15,000 female births [1,2] and one in 100,000 male births [3,4]. Typical/classical RTT is characterized by normal development up to the age of 7-18 months; then a period of developmental stagnation followed by rapid regression, deceleration of head growth, stereotypic hand movements, loss of speech and acquired motor skills. In contrast, atypical RTT refers to a subset of patients who do not meet all the criteria but manifest a variant form of the disease which exhibits heterogeneity in terms of age of onset, severity and clinical course [5,6]. Point mutations and insertion/deletion variations in *MECP2 *(Xq28) account for approximately 70-80% of cases with classic RTT [7,8] and a lower percentage of atypical cases [9-11]. Gross rearrangements in *MECP2*, which cannot be detected by sequencing or dHPLC, can be identified by a range of alternate methods such as Southern blot analysis [12,13], gene dosage assays with quantitative fluorescent PCR [14,15], and multiplex ligation-dependent probe amplification (MLPA) [16,17]. These methods contribute to unequivocal diagnosis of an additional ~10% of mutation negative cases [18]. Exons 3 and 4 in the *MECP2 *have been identified to be hotspots for rearrangements [1,2].

A limited number of twin pairs with RTT, all clinically well characterized, have also been reported in literature. Some studies have described twins showing almost concordant clinical features suggesting a genetic basis for RTT syndrome [19,20]. To the contrary, others described clinical discordance in monozygotic twins with RTT, with early developmental differences [21,22] and also with regard to seizures, scoliosis and stereotypic hand movements during adolescence in a twin pair [23]. However, genetic analysis has been reported for only in a single monozygotic twin pair [24]. This study is a second report describing the genetic basis of RTT in a 13 year old monozygotic female twin pair.

## Case presentation

The monozygotic twin females were born in an uneventful pregnancy by caesarean section with each having birth weight of 2.4 kg. The twins had normal motor milestones till about two years of age. Regression of milestones was observed following seizures, the younger of the twins at two years of age and the elder six months later. They also show minor phenotypic variation between them. The older twin had short stature (height -130 cm- < 5th Centile NCHS); and a head circumference of 51.5 cm; was non-verbal with poor response to commands; has a Vineland Social Maturity Scale (VSMS) score of 19 indicating profound mental retardation. The gait was wide based with no contractures and she had an attention span of 10-15 minutes. The younger twin also had short stature (height - 122 cm < 5th centile) and a head circumference of 48.5 cm; had a vocabulary of few single words; responded promptly to commands; as severely mentally retarded with a VSMS score of 23. She also had a wide based gait with mild knee contractures; and was on the move all the time. Both the twins had stereotypic behavior (hand biting and wringing movements); had thin and wasted limbs; had no organomegaly or evidence of head trauma or birth asphyxia; no difficulties in eating, chewing or swallowing; had normal vision; no sleep disturbances to date; no scoliosis; both were toilet trained and could indicate their needs well.

### Genetic analysis

The study was approved by the institutional ethical committee. Informed consent was obtained from the parents and blood samples (and also photographs) were collected for genetic analysis from the twin pair and parents. Patients were diagnosed as per criteria previously described [25]. Ten age and sex matched female healthy controls with no history of mental illness were also recruited from the participating hospitals. These healthy controls were used for normalization of the relative quantification assay. Fourteen highly polymorphic microsatellite repeat markers were genotyped in the patient family to confirm monozygosity.

#### a) Mutation Analysis

In order to detect point mutations and small homo-/heterozygous interstitial deletions/insertions all four exons of MECP2 were sequenced in both the twins. PCR amplification of complete exons including exon-intron boundaries were carried out using published primers [7] and the amplification products were sequenced using ABI3700 genetic analyzer.

#### b) Gene dosage analysis: detection of gene rearrangements

Exons 1 and 2 of *MECP2 *have been less frequently reported to harbor any rearrangements to date and therefore were not included for gene dosage analysis. Exons 3 and 4, which are known hotspots for rearrangements, were screened by relative quantification using Real Time PCR in conjunction with a Taqman method using an ABI Prism 7900 HT Real Time PCR system. Three probes were designed to encompass methyl-CpG binding-domain (MBD), transcriptional repression domain (TRD) and deletion prone region (DPR) of exons 3 and 4 respectively using the primer express software (Applied Biosystems) and their details are given in Table 1. RNAseP was used as an internal reference for all the experiments. PCR was performed in triplicate with a final reaction volume of 10 μl. Validation experiments for each of the three primer sets were set up with RNAseP to compare the efficiencies of the target primer with the reference primer set. Serial dilutions of a Centre d'Etude Polymorphisme Humaine (CEPH) genomic DNA control sample (1347-02) was performed with 10-fold dilutions at each step, and each dilution was run in duplicate for the standard curve calculation. Gene dosage analysis was done using the comparative Ct method [26]. Relative quantity (RelQ) was calculated as, RelQ = 2^-(DDCt)^. A RelQ ratio of around 1.5 represents three copies whereas ~0.5 represents one copy as compared to the two copies in the calibrator sample.

**Table 1:** Details of primers and probes for the Taqman Assay

Exon	Domain*	Forward Primer sequence	Reverse Primer sequence	Probe sequence
3	MBD	5' AGCGGCGCTCCATCATC 3'	5' TTCCGTGTCCAGCCTTCAG 3'	5' CATGGGTCCCCGGTCAC 3'

4	TRD	5' GCTCCTTGTCAAGATGCCTTTTC 3'	5' CCATGACCTGGGTGGATGTG 3'	5' CCCTCAGCCTTGCCC 3'

4	DPR	5' GCGTCTGCAAAGAGGAGAAGAT 3'	5' GCGGGCTGAGTCTTAGCT 3'	5' CAGCCGTCGCTCTC 3'

*Probes were labeled at 5' with FAM as reporter and at 3' with NFQ as quencher

Methyl-CpG binding-domain (MBD), Transcriptional repression domain (TRD) and Deletion prone region (DPR)

## Results

### Microsatellite markers for confirmation of monozygosity

We genotyped 14 microsatellite markers in the two parental samples, of these only nine markers were informative. These informative markers were genotyped in the twin pair. We observed identical alleles at all these nine markers confirming their monozygosity.

### Mutation screening

We screened the four exons and exon-intron boundaries among the twin pair by sequencing. No mutation was observed ruling out the involvement of any point mutations or small homo-/heterozygous interstitial deletions/insertions in the twin pair.

### Gene dosage analysis

In the absence of any mutation in the four exons of *MECP2*, we carried out gene dosage analysis to identify heterozygous deletions/duplications, if any, in the rearrangement hotspot regions of the gene. Amplification efficiencies of the target and control (RNAseP) primers were observed to be similar (~96-100% range). This is a prerequisite for using the comparative Ct method for gene dosage analysis. To determine the range of RelQ values in normal population we analyzed 10 healthy controls (5 males and females each). There was no overlap in the RelQ between the male and female controls, thus validating the use of this assay for gene dosage. RelQ values in cases deviating significantly from the controls were considered as deletions or duplications. A RelQ ratio of ~1.5 represents three copies (duplication) whereas ~0.5 represents one copy (deletion) as compared to the two copies in the female sample.

The RelQ values for the monozygotic twin pair, as expected, were found to be similar for all the three target primers (Table 2). However, both the twins showed deletion (0.60, 0.65) as compared to the normal range (0.99-1.16) of RelQ for the exon 3 probe in the MBD domain. Analysis of the parents with the same probe did not show any deviation from the normal values.

**Table 2:** RelQ values for Taqman probes in the RTT family under study

Location of Taqman Probe	Control Males	Control Females	Father	Mother	Twin1	Twin2	Result
**MBD**	0.38-0.55	0.99-1.16	0.41	1.0	**0.60**	**0.65**	**Deletion**

TRD	0.48-0.63	0.89-0.98	0.48	0.82	0.95	1.04	Normal

DPR	0.43-0.66	0.88-1.13	0.48	0.82	1.04	1.02	Normal

Methyl-CpG binding-domain (MBD), Transcriptional repression domain (TRD) and Deletion prone region (DPR)

## Conclusions

Mutations/gene rearrangements in *MECP2 *resulting in RTT are mostly germ line events resulting in sporadic cases of RTT. A small proportion of affected sibs but with unshared mutations with mother implying germline mosaicism have also been observed [13,27-29]. In addition, reports of rare cases of inherited mutations also exist [30,31]. However, in such families, the mothers showed either very mild [31] or almost unaffected [32,33] phenotype which has been explained on the basis of skewed/non-random X chromosome inactivation [33]. In the case of proven monozygotic RTT twins, shared mutation may be expected. Probability of monozygosity is > 99.9% when more than five highly polymorphic markers have identical genotypes within a twin pair [34]. Thus, 100% concordance with all the nine markers in our study confirms the monozygotic origin of the twin pair.

Mutation screening of the four exons of *MECP2 *in the twin pair did not show any point mutation. However, both monozygotic twins showed a heterozygous deletion in exon3 identified by Taqman assay (Table 2). The rearrangement was not shared with either of the parents, thus representing a *de novo *origin of the probable pathological variation in the family. As mentioned earlier, there were minor phenotypic differences in these twins. This may either be due to non-random (in)activation of the paternal/maternal X chromosomes [22] or may be due to yet poorly understood epigenetic signatures, if any, at MECP2 locus. The former assumption has partly been proved by another study where the twin sisters shared the mutation (R294X), but showed discordant clinical phenotype; they observed skewing in favour of the paternal allele in the twin with more severe phenotype [24]. Preferential paternal X chromosome involvement has been shown in several other sporadic RTT cases [35,36]. Twin studies may be useful to understand the genetic as well as the non-genetic contribution to the disease phenotype. Discordance witnessed among some twin pairs including the pair described in this study warrant further studies to unravel epigenetic mechanisms influencing RTT phenotype.

## Consent

Written informed consent was obtained from the parents of the patients for publication of this case report and any accompanying images. A copy of the written consent is available for review by the Editor-in-Chief of this journal.

## Competing interests

The authors declare that they have no competing interests.

## Authors' contributions

All authors have read and approved the final manuscript. KM was involved in the study design, performed molecular genetic and statistical analyses, compiled the data and wrote the manuscript; MK was the principal clinical investigator involved in study design, defining exclusion and inclusion criteria of study subjects and were mainly responsible for identification of the study subjects from their respective clinical centre; RJ participated in its design and coordination and drafting the manuscript. TBK was the principal geneticist and coordinator of the project, involved in conceptualization of the project, study design, supervising genetic analyses and preparation of the manuscript.

## Pre-publication history

The pre-publication history for this paper can be accessed here:

http://www.biomedcentral.com/1471-2350/12/113/prepub
